# The hybrid crosslinking method improved the stability and anti-calcification properties of the bioprosthetic heart valves

**DOI:** 10.3389/fbioe.2022.1008664

**Published:** 2022-09-09

**Authors:** Yuhong Liu, Zhongshi Wu, Chunyang Chen, Ting Lu, Mingzhe Song, Xiaoke Qi, Zhenlin Jiang, Sixi Liu, Zhenjie Tang

**Affiliations:** ^1^ Department of Cardiovascular Surgery, The Second Xiangya Hospital of Central South University, Changsha, Hunan, China; ^2^ Engineering Laboratory of Human Province for Cardiovascular Biomaterials, Changsha, Hunan, China

**Keywords:** bioprosthetic heart valves, bovine pericardium, glutaraldehyde, hybrid cross-linking, stability, anti-calcification

## Abstract

The bioprosthetic heart valves (BHVs) are the best option for the treatment of valvular heart disease. Glutaraldehyde (Glut) is commonly used as the golden standard reagent for the crosslinking of BHVs. However, the obvious defects of Glut, including residual aldehyde toxicity, degradation and calcification, increase the probability of valve failure *in vivo* and motivated the exploration of alternatives. Thus, the aim of this study is to develop a non-glutaraldehyde hybrid cross-linking method composed of Neomycin Trisulfate, Polyethylene glycol diglycidyl ether and Tannic acid as a substitute for Glut, which was proven to reduce calcification, degradation, inflammation of the biomaterial. Evaluations of the crosslinked bovine pericardial included histological and ultrastructural characterization, biomechanical performance, biocompatibility and structural stability test, and *in vivo* anti-inflammation and anti-calcification assay by subcutaneous implantation in juvenile Sprague Dawley rats. The results revealed that the hybrid crosslinked bovine pericardial were superior to Glut crosslinked biomaterial in terms of better hydrophilicity, thermodynamics stability, hemocompatibility and cytocompatibility, higher Young’s Modulus, better stability and resistance to enzymatic hydrolysis, and lower inflammation, degradation and calcification levels in subcutaneous implants. Considering all above performances, it indicates that the hybrid cross-linking method is appropriate to replace Glut as the method for BHV preparation, and particularly this hybrid crosslinked biomaterials may be a promising candidate for next-generation BHVs.

## 1 Introduction

As the population continues aging, valvular heart disease, as a common kind of cardiovascular disease, has becomes a serious danger to human health all over the world (Emmert et al., 2018; Li, 2019). Valve replacement is still the gold standard of treatment for end-stage valvular disease and extends the lifespan of patients (Mohammadi and Mequanint, 2011). Nearly three hundred thousand patients received valve replacement surgery each year in recent decades, and this number is estimated to be more than triple by 2050 (Fioretta et al., 2018). Currently, there are two major types of commercial valve substitutes: mechanical heart valves (MHVs) and bioprosthetic heart valves (BHVs). MHVs are not suitable for everyone because of the disadvantages of long-term anti-coagulation and poor hemodynamic, although they can be used for a longer period after operation. Compared with MHVs, besides the better biocompatibility and hemodynamics, BHVs can also be suitable for transcatheter heart valve replacement (THVR) which expanded the usage of BHVs in clinical applications (Auffret et al., 2017; Lim, 2018; Catterall et al., 2020). However, current clinical BHVs suffer from many defects such as poor durability, leaflet damage, and calcium deposition, therefore, it is urgent to enhance these properties of BHVs (Lopez-Moya et al., 2018; Zhang et al., 2018).

The native heart valve have evolved into multilayered leaflet structures, such as fibrosa, spongiosa, and ventricularis. These layers are mainly composed of collagen, elastin, proteoglycans, and glycosaminoglycans. The fibrosa layer is mainly composed of a dense collagen fiber network, which is considered to be the major stress bearing layer. The spongiosa layer contains high concentration of glycosaminoglycans and proteoglycans. While the ventricularis layer consists of a dense network of elastin and collagen fibers (Schoen, 1997; Ayoub et al., 2016). Admittedly, valves experience a complex range of forces and dynamic behaviors that include surface shear from blood flow, tensile loading during closure, and flexure as the valves transition between the open and closed state (Lovekamp et al., 2006; Sacks et al., 2009). The long-term stability of each component is fundamental to maintaining the function of heart valve.

However, the currently commercially available BHVs are all cross-linked by Glutaraldehyde (Glut), which crosslinked with collagen through reversible Schiff bases and still cannot prevent the degradation of the collagen triple helix structure (Zilla et al., 2008; Bezuidenhout et al., 2009). The previous study also showed that the bending stiffness of the cups after Glutaraldehyde treatment changed during the cyclic fatigue test, indicating the degradation of the ECM structure (Deshmukh et al., 1971). Glut only cross-links collagen and has no effect on elastin and glycosaminoglycans (GAGs) which may play a vital role in valve durability. Elastin and GAGs lack of lysine-derived amino groups which are needed for Glut cross-linking. *In vivo*, the loss of GAGs and the denaturation and degradation of elastin further lead to the deposition calcification of BHVs (Isenburg et al., 2004; Mercuri et al., 2007). In addition, the loss of GAGs can lead to poor biomechanical properties including increased tissue buckling and decreased flexural rigidity (Vyavahare et al., 1999). Furthermore, the residual aldehyde groups might also be toxic to host cells, leading to calcification and induce inflammation which is another problem of Glut cross-linked BHVs (Kumar et al., 2013).

To prepare BHVs with better performance, some non-glutaraldehyde cross-linking agents are used to stabilize extracellular matrix, such as 1-ethyl-3-(3-dimethylaminopropyl) carbodiimide hydrochloride (EDC), Genipin, Tannic acid (TA), Epoxides, Neomycin trisulfate (NE), Gelatin methacryloyl, and so forth. Most crosslinking methods use one or two agents to stabilize two of the main components of BHVs (Sung et al., 1996a; Tripi and Vyavahare, 2014). However, whether these methods are better than Glut is still questionable, but so far, none has been put in clinical application (Guo et al., 2018). Therefore, the simultaneous stabilization of three fundamental components is crucial to prolong the durability of BHVs. Based on previous studies, we selected three reagents with definite cross-linking effects on main components of the extracellular matrix and without toxicity. Neomycin trisulfate, a potent hyaluronidase inhibitor, has previously been shown to inhibit GAG loss in BHV (Friebe et al., 2011; Leong et al., 2013). Polyethylene glycol diglycidyl ether (PE) belongs to epoxy compound with multiple reactive epoxy groups, and was confirmed to be non-toxic and react with the amino and carboxyl groups of collagen, which can increase the strength, thermal and biological stability of biomaterials (Lohre et al., 1993; Sung et al., 1997). Tannic acid is a plant polyphenol belonging to the galloyl-glucose family and is a proved elastin stabilizer (Isenburg et al., 2005). We hypothesized to develop a novel hybrid crosslinked method (NPTD) that utilizes Neomycin trisulfate, Polyethylene glycol diglycidyl ether and Tannic acid to stabilize three fundamental extracellular matrix components could improve the biomechanics, biocompatibility, anti-degradation and anti-calcification properties of BHVs. Then the effect of hybrid crosslinking on the bovine pericardium (BP) was assessed, including the biomechanical properties, ultrastructure, anti-degradation and biocompatibility *in vitro*. In addition, the inflammation, stability and calcification characteristics were further explored through a rat subcutaneous implantation model. To our knowledge, this is the first time to demonstrate that this hybrid crosslinking method stabilized the main components of bovine pericardium as well as improved the stability and anti-calcification of tissue.

## 2 Materials and methods

**FIGURE 1:**
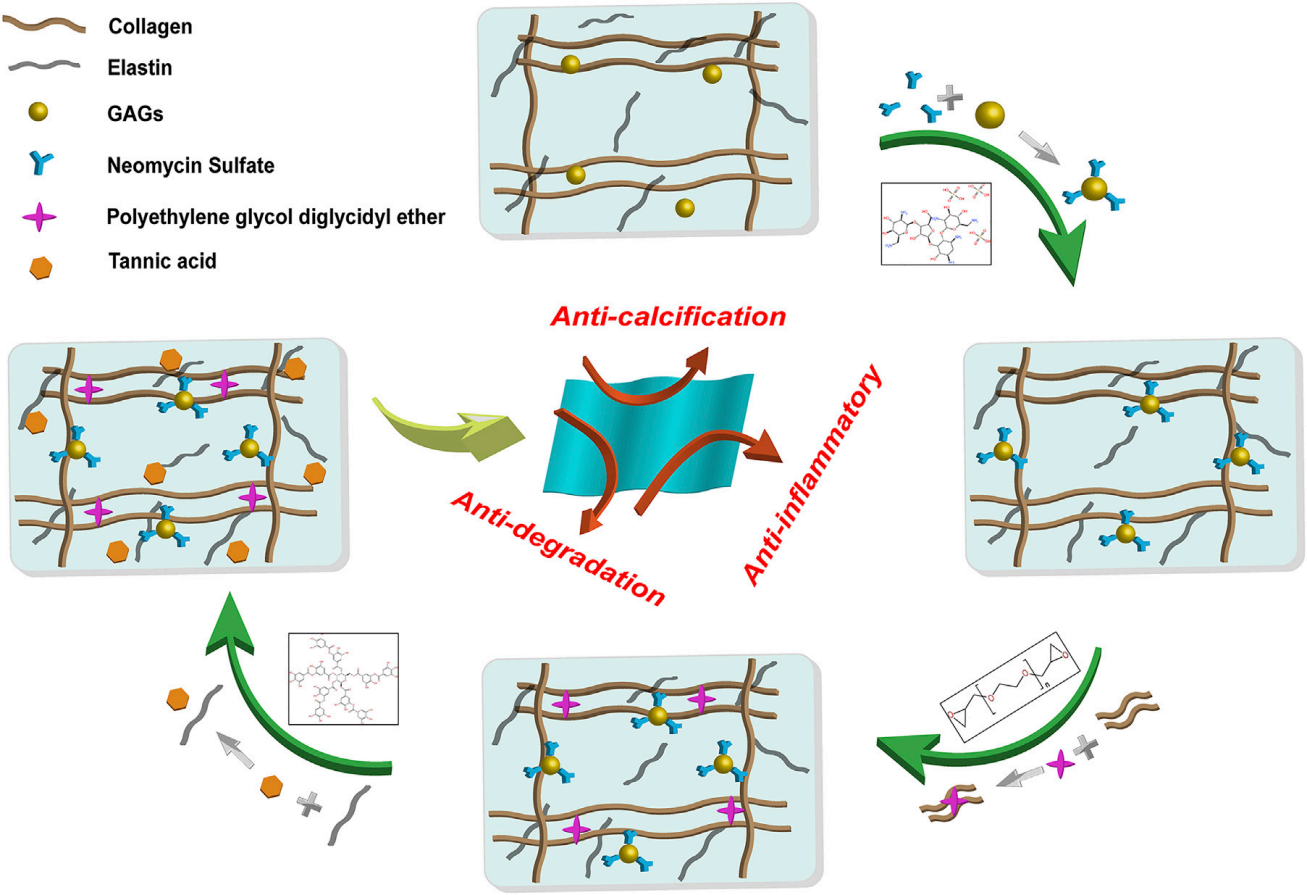
General procedure and reaction principle of the preparation of the hybrid crosslinked bovine pericardium. The method was followed by Neomycin to stabilize GAGs, PE to stabilize collagen fibers, and Tannic acid to stabilize elastin. GAGs: Glycosaminoglycans. PE: Polyethylene glycol diglycidyl ether.

Yellow cattle pericardium with warm ischemia time less than 30 min were obtained from local slaughterhouse, stripped off the residual fat and tissue and rinsed with phosphate buffered saline (PBS). The pericardium was cut into 10 cm × 10 cm leaflets and stored at 4°C in PBS containing 1% penicillin/streptomycin. Thereafter, the leaflets were further processed as indicated in our scheme (Figure 1).

### 2.1 Crosslinking procedure

The pericardium leaflets were divided into two groups with different crosslinking methods.

Glut crosslinked leaflets were placed in 0.6% GLUT, 50 mM HEPES buffered saline solution at pH 7.4, ambient temperature. After 24 h, the solution was replaced by 0.2% GLUT solution for an additional 6 days (Tripi and Vyavahare, 2014) (GA samples).

The procedure of crosslinked with Neomycin (NE), Polyethylene glycol bisglycidyl ether (PE) and Tannic acid (NPTD samples).

The leaflets were incubated in a solution of 1 mM neomycin trisulfate in 50 mM MES buffered solution at pH 5.5, ambient temperature under constant orbital shaking for 1 h. The solution was decanted and leaflets were then incubated in a 30 mM 1-ethyl-3-(3-dimethylaminopropyl) carbodiimide (EDC) and 6 mM N-hydroxysuccinimide (NHS) in 50 mM MES buffered saline (pH 5.5) for 24 hours. The solution was decanted and leaflets were then incubated in a 4% PE solution (0.1 M PBS buffer, pH 4.5, at 4°C for 2 days, 25°C for 1 day, 37°C for 1 day, 56°C for 1 day and with the one change every 2 days. Then adjusted PH 9.5, at 56°C for 1 day) for 6 days. Then further treated with 0.8% tannic acid (0.1 M PBS buffer, pH 5.5, ambient temperature for 3 days). Finally, the crosslinked pericardium was dehydrated with 60% and 70% alcohol for 1 day and stored in 4°C PBS containing 1% penicillin/streptomycin. The leaflets were sterilized by irradiation (radiation dose: 25 KGy).

### 2.2 Histological characterization

To observe the histological morphology of the pericardium after cross-linked, the hematoxylin and eosin (HE, Servicebio, G1005) staining, Masson’s trichrome (Servicebio, G1006) staining, elastic Van-Gieson (EVG, Servicebio, GP1035) staining and Alcian blue staining were executed. The fresh pericardium (NA), Glut cross-linked (GA), and the hybrid cross-linked (NPTD) samples were fixed with paraformaldehyde, dehydrated in graded alcohols, and then embedded in paraffin and sectioned (5 μm). The samples were observed using an optical microscope (Nikon, NIS-eLEMENTS d).

### 2.3 Ultrastructural and hydrophilic characterization

For scanning electron microscopy (SEM) (Liu et al., 2022), the NA/GA/NPTD samples were fixed and dried by a graded ethanol series (30%, 50%, 70%, 90%, 100%), then freeze-dried for dehydration. The surface and transection of the dried samples were sputter coated with 20 nm gold and observed with a FEI Nova NanoSEM (FEI Electron Optics B.V, Czech).

The hydrophilicity of the three groups was characterized by the contact angle test. The fresh or cross-linked pericardium leaflets (1 cm × 1 cm) with a flat surface were fixed and freeze-dried (*n* = 8). Furthermore, the test was carried on an optical contact angle measuring instrument (SDC-200S, ShengDing, China).

### 2.4 Differential scanning calorimetry and biomechanical properties

The thermal denaturation temperature (Td) of collagen was evaluated by differential scanning calorimetry (DSC, 204F1, NETZSCH, Germany) measurement (Mercuri et al., 2007). Briefly, small pieces of pericardium leaflets (6–10 mg) were cut from the same region, freeze-dried and placed in the sealed pans. The samples were equilibrated at 20°C and heated at 10°C/min up to 120°C under N2 atmosphere. The Td was recorded as the maximum value of the end other M peak.

The tensile testing was performed on an INSTRON instrument (3343, Instron, United States) (Liu et al., 2020). The three groups of NA/GA/NPTD were cut into a 6 cm by 1 cm strip shape (*n* = 6). Before the test, the samples were spread in PBS buffer and the average thickness was calculated by measuring the thickness of four random points on each sample. A tensile rate of 10 mm/min and 200 N sensor was exerted on the leaflets until invalidation. The Young’s modulus, Maximum tensile stress and Maximum load were measured.

### 2.5 Cytocompatibility analysis of leaflets

#### 2.5.1 Cytotoxicity assessment

Prior to cell culturing (Liu et al., 2022), the leaflets were cut into a square shape with 1 cm^2^ × 1 cm^2^ and cultured in high glucose Dulbecco’s Modified Eagle Medium with 10% fetal bovine serum (DMEM/10%FBS, Servicebio, G4510, G8001, China) at 37°C for 24 h at a density of 2.5 ml/cm^2^. Then the leach liquor were collected. Human umbilical vein endothelial cell line (EAhy926) was cultured in DMEM/10%FBS. The medium was replaced by DMEM/10% FBS and leach liquor diluted 1:2. 5,000 cells were seeded in 96 well plates (*n* = 6) with 200 μl medium. Cells were maintained in culture at 37°C with 5% CO_2_ for 1, 3, and 5 days. Negative controls were prepared with DMEM/10% FBS alone. The mitochondrial metabolic (MTT, Servicebio, G4104, China) was used to evaluate cell growth and determine the optical density at 570 nm with a microplate reader (Thermo Scientific, Multiskan Sky). Relative growth rate (RGR) was used to assess cytotoxicity in each group, RGR = (mean OD for each group)/(mean OD of the negative control) × 100% (Xu et al., 2017).

#### 2.5.2 Cell proliferation and viability on leaflets

γ-Ray sterilized leaflets of the NA, GA and NPTD (5 mm × 5 mm) were rinsed three times with PBS and incubated with DMEM/10% FBS cell culture medium for 24 h. The leaflets were placed into a 48-well plate and Human umbilical vein endothelial cell (HUVECs) were seeded on the leaflets at a density of 10,000 cells/well. After 5 days of incubation, the leaflets were transferred into another 48-well plate, 100 μl of Carboxy-2′,7′-dichlorofluorescein diacetate live cell fluorescent dye (6-CDCFDA) working solution was added and were placed in a cell incubator for 30 min, then visualized under a fluorescence microscope (Nikon, NIS-eLEMENTS d) (Liu et al., 2022).

### 2.6 Hemocompatibility analysis of leaflets

Platelet adhesion assay was conducted to evaluate the platelets behaviors of each sample (Liu et al., 2019). Fresh whole blood collected from rabbit was citrated with sodium citrate (1:9). Platelet-rich plasma (PRP) was prepared by centrifugation of the citrated blood at 1,100 rpm for 10 min at 4°C. The pericardium leaflets (*d* = 8 mm) were placed into 48-well plates and incubated with PBS for 2 h at 37°C, followed by replacing with 300 μl fresh PRP and incubating for 1 h at 37°C. After then, the PRP was discarded and the samples were washed with PBS three times. For SEM observation, the pericardium leaflets were fixed with paraformaldehyde for 2 h Then the leaflets were followed by gradient dehydration and lyophilization as mentioned before. Furthermore, for Lactate dehydrogenase (LDH) assay (Sivaraman and Latour, 2010), the pericardium leaflets lysed with 0.5% Triton X-100 (v/v) for 30 min at room temperature. The supernatant was collected and detected by LDH kit (A020, JIANCHENG, China) according to the manuals.

Hemolysis test was primarily conducted to evaluate the hemocompatibility of the samples (Hu et al., 2021). Fresh whole rabbit blood was centrifuged (1,100 rpm, 10 min) at 4°C. The sedimental red blood cells (RBCs) were washed with normal saline (NS) three times. A centrifugation of 5 min at 3,700 rpm was performed after each wash. The RBC concentrate was diluted to a final concentration of 5 vol% with NS. The samples were immersed in 1 ml 5% RBCs suspension and incubated at 37°C for 1 h. The 300 μl NS (0% lysis) and 300 μl 1% SDS (82% lysis) were added to the RBC suspension and were used as the negative and positive controls, respectively. After centrifugation at 1,100 rpm for 10 min, the absorbance of the supernatant at 540 nm was detected by a microplate reader. To determine the hemolytic ratio, the follow equation was used.

Hemolytic ratio = (sample − negative control)/(positive control − negative control).

### 2.7 *In vitro* enzymatic challenge studies

To test the resistance to collagenase, elastase and hyaluronidase of the three groups (Tam et al., 2015). The pericardium were cut into 1 cm × 1 cm leaflets, rinsed in deionized water, frozen, lyophilized, and weighed. The samples were incubated in 1.5 ml of 200 U/ml collagenase (Type I, Sigma, C9891, United States) in 50 mM TES, 0.36 mM CaCl2, (pH 7.4) for 24 h, or 25 U/ml Elastase (Biomatik, A4126, China) in 50 mM Tris, 10 mM CaCl2, (pH 7.8) for 24 h at 37°C while shaking at 150 RPM, The digested samples rinsed in deionized water, frozen, lyophilized, and weighed. Percent weight loss was calculated. For resistance to GAGases, the samples were incubated in 1.5 ml of 5 U/ml hyaluronidase (Sigma, H3506, United States) in 0.02 M phosphate buffer, (pH 5.35) for 24 h at 37°C while shaking at 150 RPM. Then, the samples were washed in deionized water, frozen, and lyophilized. Total GAGs content was measured using the GAGs assay kit (GENMED SCIENTIFICS INC., GMS50655. United States).

### 2.8 Storage studies

After irradiation sterilization treatment, the samples (1 cm × 1 cm, *n* = 8) were soaked in 30 ml PBS in a sterile environment (Liu et al., 2020), and then uniformly oscillated at 37°C for 90 days (100 rpm/min). The solution was replaced every 2 weeks by sterile PBS. When reaching the preset time point, the sample was thoroughly washed three times with ultrapure water. Then, a part of the sample was fixed with paraformaldehyde and saved for histological analysis. The other part was freeze-dried as soon as possible for further collagen (BIOCOLOR, S2000, United Kingdom), elastin (BIOCOLOR, F2000, United Kingdom)and GAGs quantification assay.

### 2.9 *In vivo* subcutaneous implantation

All animal experiments were approved by the Institutional Animal Care and Use Committee (IACUC), The Second Xiangya Hospital, Central South University, China. All operations were conducted while keeping with the Guide for Care and Use of Laboratory Animals. The calcification and immune inflammation responses against the different groups of NA/GA/NPTD were evaluated in rat subcutaneous implantation models (Qi et al., 2022). Before the *in vivo* experiment, 1 cm^2^ × 1 cm^2^ samples were sterilized by irradiation treatment. Subcutaneous implantation was performed on male juvenile Sprague Dawley rats (SD, *n* = 20) anesthetized by sodium pentobarbital (30 mg/kg). A longitudinal incision was made on the back of the rat with sterile scissors. The pericardial samples were placed as flat as possible in the subcutaneous pocket, and the incision was closed with 2-0 Mersilk suture. For the immune inflammatory response, three groups of leaflets were implanted into rats, and the samples (*n* = 4) were retrieved at 1 week, 3 weeks after implantation. For calcification, the samples (*n* = 8) were retrieved after 90 days of implantation. Then, a part of the sample was fixed with paraformaldehyde and saved for histological and immunohistochemical analysis. The other part was frozen at −80°C as soon as possible for further calcium deposition assay and elastin and GAGs quantification assay as mentioned before.

#### 2.9.1 Immunohistochemical analysis

The Immunohistochemistry (IHC) staining for CD68 and CD3 was performed on specimens implanted at 1 week and 3 weeks *in vivo*, the sections (5 μm) were deparaffinized, rehydrated and processed for antigen retrieval (Heat induced antigen retrieval was performed in 0.01 M citric acid buffer with high temperature and pressure for 3 min). Then the sections were incubated with primary antibodies at 4°C overnight. Rabbit anti-rat CD68 antibody (dilution 1:400; Servicebio, GB11067) was used to label the macrophage cells, and rabbit anti-rat CD3 antibody (dilution 1:700; Servicebio, GB11014) was used to label the T cells. The number of inflammatory cells observed in the specimens of each group was quantified by counting. At least five fields were counted for each specimen.

#### 2.9.2 Histological and quantification analysis

The fixed implants of each group of samples were gradient dehydrated, then embedded in paraffin and sectioned (5 μm) for light microscopy analysis. H/E stain, Masson’s trichrome, EVG and Alcian blue stains were used to visualize and assess the cell infiltration, fiber distribution, degradation of elastin and preservation of GAGs. Alizarin red stains were used to visualize and assess the distribution of the calcium deposition in the implanted samples. In addition, for specimens implanted *in vivo* for 90 days, the preservation of elastin and GAGs was determined using quantitation kits as previously described.

#### 2.9.3 Calcium deposition assay

Briefly, the wrapped tissues were removed by tweezers carefully from the freshly harvested samples. After which the obtained tissues were lyophilized, weighted and hydrolyzed with 6 M HCl for 24 h at 96°C. The supernatants were filtered and diluted as 1:20 in deionized water. Calcium content were analyzed using the atomic adsorption spectrometry (Spectro Analytical Instruments, Thermo Fisher, United States) (Qi et al., 2022). The calcium contents were normalized to the dry weights of the implanted samples.

### 2.10 Statistical analysis

The results of quantitative studies were expressed as the median and interquartile range or the mean ± standard error of the mean (SEM). The Shapiro—Wilk normality test was used to define whether data were normally distributed. One-way analysis of variance (ANOVA) was conducted to determine differences between three groups for continuous variables with normal distribution. The Kruskal—Wallis test with Tukey’s post hoc test was conducted to evaluate the differences for non-normally distributed data. All the statistical analyses were performed with GraphPad Prism 8.4.0 (GraphPad Software, United States). *p*-values < 0.05 were considered to be statistically significant.

## 3 Results

### 3.1 Histological and ultrastructural characterization of leaflets

**FIGURE 2:**
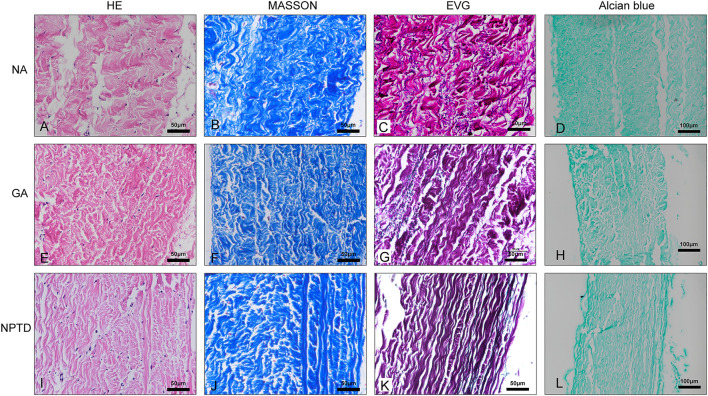
Histological staining images of the bovine pericardium. The structure of the leaflets remained intact and compact after the GA and NPTD crosslinked compared to the natural pericardium. The images show the HE staining **(A,E,I)**, Masson’s trichrome staining **(B,F,J)**, elastic Van-Gieson staining **(C,G,K)** and Alcian blue staining **(D,H,L)**.

**FIGURE 3:**
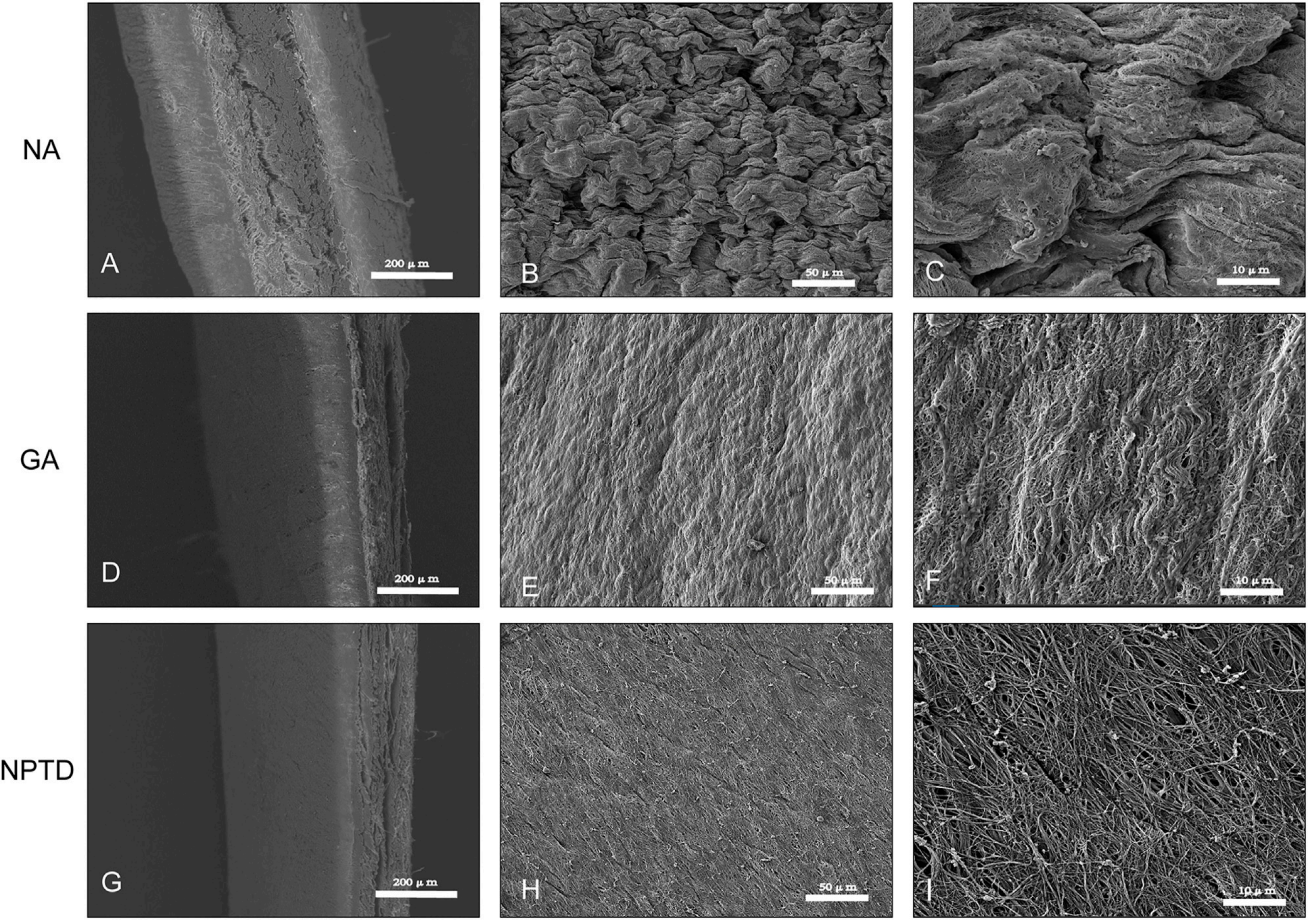
SEM micrograph of the surface and transection of the bovine pericardium. The tissue feature of the NA was the loose porous and rough **(A,B,C)**. The feature of flat and compact structure in the NPTD group **(G,H,I)** was similar to that of the GA group **(D,E,F)**.

**FIGURE 4:**
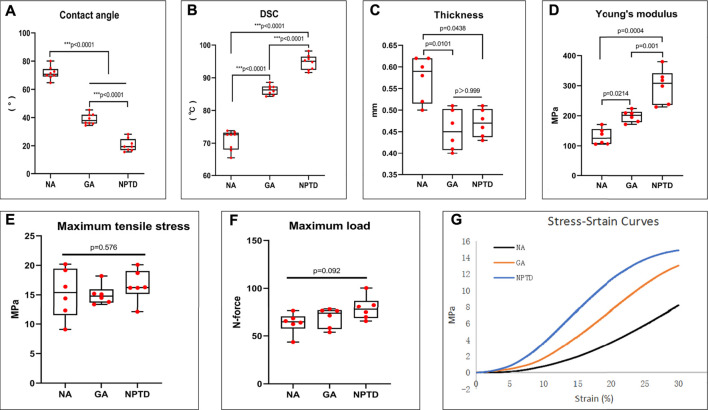
The water contact angle, thermal denaturation temperature (Td) and biomechanical properties of the bovine pericardium. The water contact angle of the leaflets decreased significantly after NPTD crosslinked compared with those of the NA and GA groups **(A)**. Td of the NPTD samples was distinctly improved compared to those of the NA and GA samples **(B)**. The thinness of the leaflets decreased after NPTD crosslinked and were close to those of the GA group **(C)**. The Young’s modulus was significantly improved in the NPTD group compared with the GA group **(D)**. The Maximun Tensile Stress and Maximum Load among the three groups was no significant difference **(E,F)**. The representative stress/strain curves of the groups **(G)**. Statistical analysis was performed by Kruskal–Wallis test with Tukey’s post hoc test.

To observe the surface and histological characteristics of crosslinked bovine pericardium, the pericardium was analyzed by histological staining, SEM, and water contact angle. The structure of the extracellular matrix was visualized by HE, Masson’s trichrome, EVG and Alcian blue staining (Figure 2). After crosslinking, The GAGs was preserved well, the collagen fiber becomes more condensed, elastic fiber had better integrity in the NPTD and GA groups. From the results of SEM (Figure 3), the cross-linked pericardial surface was flat with stretched fibers and a condense structure. The images of transection also indicated that the GA and NPTD groups had a flat surface and a compact structure. The hydrophilicity results showed that the water contact angle of the fresh pericardium was approximately 80° degrees, the GA group was 40° and the NPTD group was 25° (Figure 4A). The water contact angle of the NPTD group was significantly lower than that of the NA group and GA group, indicating that the NPTD group has better hydrophilicity.

### 3.2 Biomechanical properties of leaflets

To evaluate the collagen stability of the crosslinked pericardium, the thermal denaturation temperature of the samples was determined by DSC. As shown in Figure 4B, the thermal shrinkage temperature of the NPTD (94.6 ± 0.8°C) group was significantly higher than that of the NA (70.8 ± 1.1°C) and GA (86.3 ± 0.5°C) groups. The above results demonstrated that the hybrid crosslinking method could effectively crosslink the collagen fiber and improve the collagen stability of bovine pericardium.

Furthermore, the tensile testing was carried out to evaluate the mechanical properties of the crosslinked pericardium. The thickness of the NA (0.57 ± 0.02 mm) group was significantly higher than that of the GA (0.45 ± 0.02 mm) and NPTD (0.47 ± 0.01 mm) groups (Figure 4C). The Young’s Modulus (YM) of the NPTD (298.3 ± 23.4 MPa) was significantly higher than that of the GA (197.6 ± 8.0 MPa) and NA (130.6 ± 11.2 MPa) groups (Figure 4D). Meantime, there was no significant difference in the Maximun Tensile Stress and Maximum Load among the three groups (Figures 4E,F). The representative stress strain curves of each group were shown in Figure 4G.

### 3.3 The cytotoxic and cell viability on leaflets

**FIGURE 5:**
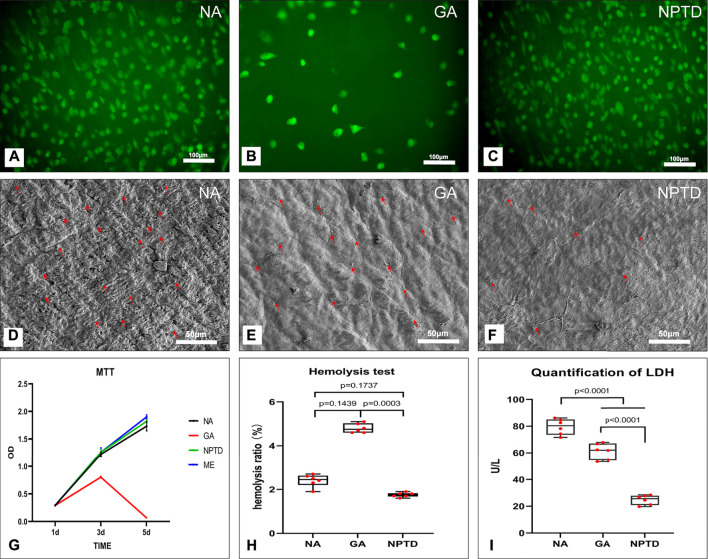
The cytocompatibility and Hemocompatibility of the bovine pericardium. The human umbilical vein endothelial cells (HUVECs) proliferation and morphology was assessed by 6-CDCFDA live cell fluorescent staining **(A,B,C)**, the HUVECs almost completely covered the surface of the NA and NPTD groups on day 5 while only a few cells in the GA group **(A,B,C)**. The cytotoxicity of the leaflets was determined by MTT assay, the relative growth ratios of GA group was significantly lower than that of the NA and NPTD groups **(G)**. The platelet adhesion on the surface of the samples was evaluated by SEM imaging **(D,E,F)** and LDH assay **(I)**. Red arrows indicate adherent platelets. The quantification of LDH in the NPTD group was significantly lower than that of the NA and GA groups. The hemolysis ratio of LDH in the NPTD and NA group was significantly lower than that of the GA group **(H)**. Statistical analysis was performed by one-way analysis of variance (ANOVA).

The HUVECs was used to evaluate the toxicity and viability of crosslinked leaflets. The cytotoxicity of each group was determined by MTT assay. As shown in Figure 5G, the relative growth ratios (RGRs) of cells were grown in the leach liquor from the pericardium of each group after 1, 3, and 5 days of culture were evaluated. On day 1, no apparent differences were observed between the four groups; on day 3 and day 5, the RGRs of GA group was significantly lower than that of the NA and NPTD groups. In addition, on day 5, the cells in the GA group were almost inviable.

The proliferation and viability of HUVECs on the NA/GA/NPTD groups *via* the live cell 6-CDCFDA fluorescent staining. As shown in Figure 5, after 5 days of cultured, the cell number on the surface of the NA and NPTD groups increased over time whereas the number of cells of the GA groups did not increase. HUVECs almost completely covered the surface of the NA and NPTD groups on day 5, in comparison, only a few cells could be identified on the surface of the GA group (Figures 5A–C).

### 3.4 Hemocompatibility of the leaflets

To evaluate the hemocompatibility of the crosslinked pericardium, the hemolysis ratio, platelet adhesion and LDH activity were investigated. The platelet adhesion on the surface of the samples was evaluated by SEM imaging (Figures 5D–F) and LDH assay (Figures 5I). The SEM images of the platelet adherent to the three groups’ surfaces. The surface of the NA and GA groups were covered by a large number of spreading platelets (Figure 5C). By contrast, a small number of platelets were observed on the surface of the NPTD group. Furthermore, the quantification of LDH in the NPTD group was significantly lower than that of the NA and GA groups.

Meanwhile, As shown in Figure 5H, the hemolysis tests of the samples showed that the hemolysis rates of the NA and NPTD groups were both <2%, which met the safety standards of blood contact materials (hemolysis rate <5%). However, the hemolysis rate of the GA group (4.8 ± 0.09%) was significantly higher than the NPTD groups.

### 3.5 Investigation of resistance to enzymatic hydrolysis

**FIGURE 6:**
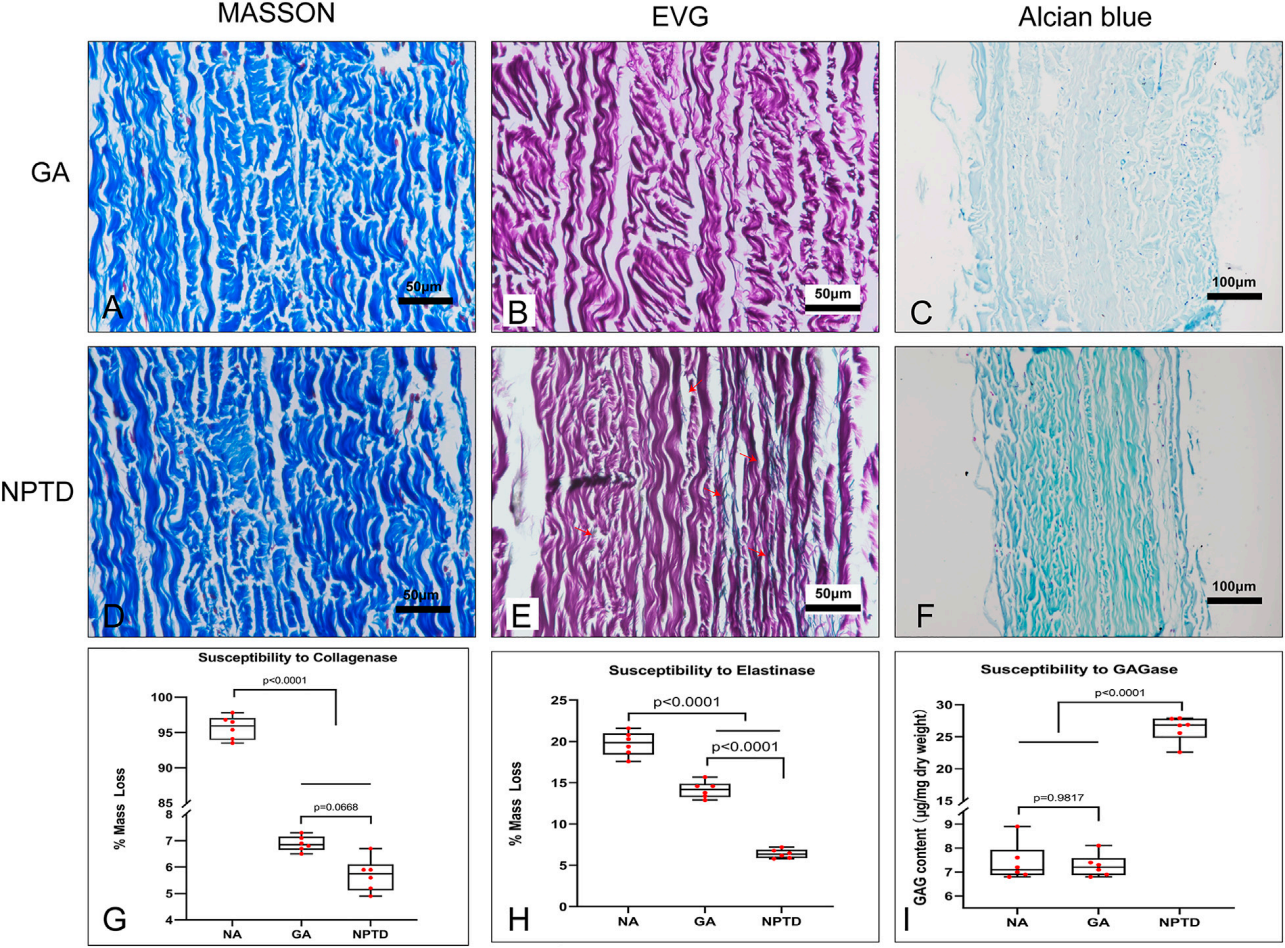
Histological features and quantitative analysis after enzymatic hydrolysis of cross-linked pericardium. The images of Masson’s trichrome staining after collagenase hydrolysis **(A,D)**. The images of elastic Van-Gieson staining after elastinase hydrolysis **(B,E)**, Red arrows show preserved elastic fibers in the NPTD group. The images of Alcian blue staining after hyaluronidase hydrolysis **(C,F)**. The quantitative analysis of mass loss and residual GAGs content in each group after enzymatic hydrolysis **(G–I)**. Statistical analysis was performed by one-way analysis of variance (ANOVA).

The collagenase, elastase and hyaluronidase were used to evaluate the resistance of three main components of crosslinked pericardium to enzymatic hydrolysis. Each group of samples had a loss of mass when treated with collagenase or elastase. Samples from the NA group were almost completely dissolved after collagenase treatment for 24 h, and the relative loss of mass was more than 95%. The Masson’s trichrome staining showed that the structure of collagen fibers in the GA and NPTD groups remained compact with treatment of collagenase (Figures 6A,D). In addition, there was no difference in the relative mass loss between the GA (6.88 ± 0.12%) and NPTD (5.70 ± 0.25%) groups, but it was significantly lower than that in the NA group (Figure 6G). After treatment of elastase, the EVG staining showed that there were many elastic fibers were still visible in the NPTD group compared with the GA group (Figure 6 B,E). Furthermore, the relative mass loss was significantly lower in the NPTD (6.41 ± 0.22%) group compared with the GA (14.17 ± 0.41%) and NA (19.73 ± 0.56%) groups in the treatment of elastase (Figure 6H). After hyaluronidase hydrolysis, Alcian blue staining showed that the blue color of the NPTD group was similar to that before enzymatic hydrolysis, while GA group was lighter (Figures 6C,F). Meanwhile, the content of GAGs was significantly higher in the NPTD (26.27 ± 0.81 μg/mg) group than that in the GA (7.23 ± 0.19 μg/mg) and NA (7.40 ± 0.32 μg/mg) groups (Figure 6I).

### 3.6 Stability of leaflets *in vitro*

**FIGURE 7:**
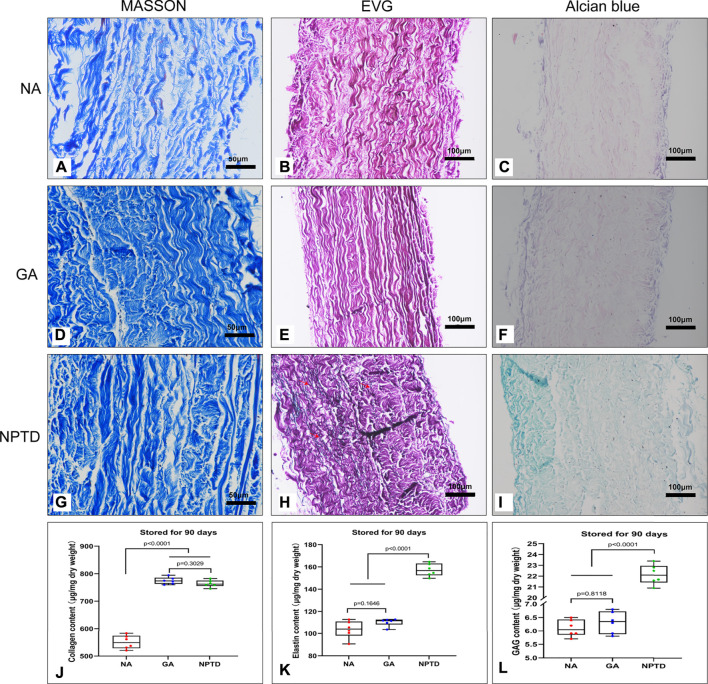
The stability of the pericardium after 90 days of storage *in vitro*. The images show the Masson’s trichrome staining **(A,D,G)**, elastic Van-Gieson staining **(B,E,H)** and Alcian blue staining **(C,F,I)**. Red arrows show preserved elastic fibers in the NPTD group. The residual content of collagen in the GA and NPTD groups was significantly higher than that in the NA group **(J)**. The residual content of elastin and GAGs in the NPTD groups was significantly higher than that in the NA and GA group **(K,L)**. Statistical analysis was performed by one-way analysis of variance (ANOVA).

To investigate the storage stability of the cross-linked pericardium *in vitro*, we stored the pericardium of each group in PBS for 90 days on a shaker at 37°C. The stability of the samples was analyzed by Masson’s trichrome, EVG, Alcian blue staining and the residual contents of collagen, elastin and GAGs (Figure 7). In both GA and NPTD groups, the structure of the collagen remained compact, and the residual content of collagen in the GA (774.75 ± 5.11 μg/mg) and NPTD (763.98 ± 5.12 μg/mg) groups was not significantly different, but was significantly higher than that in the NA (550.27 ± 10.02 μg/mg) group (Figures 7A,D,G,J). In the NPTD group, black elastic fibers were still visible in EVG staining, but few in the GA and NA groups (Figures 7B,E,H). In addition, the residual elastin content of NPTD group (157.28 ± 2.31 μg/mg) was also significantly higher than that of the GA (110.38 ± 1.38 μg/mg) and NA (103.75 ± 2.23 μg/mg) groups (Figure 7K). Furthermore, the Alcian blue staining showed that the GAGs of NPTD could still be stained blue, while there was no obvious blue in the GA and NA groups (Figures 7C,F,I). The content of GAGs was similar to that of elastin in the NPTD group (22.15 ± 0.37 μg/mg), which was significantly higher than that of the GA (6.32 ± 0.17 μg/mg) and NA (6.10 ± 0.13 μg/mg) groups after 90 days of storage (Figure 7L).

### 3.7 Inflammation response *in vivo*

**FIGURE 8:**
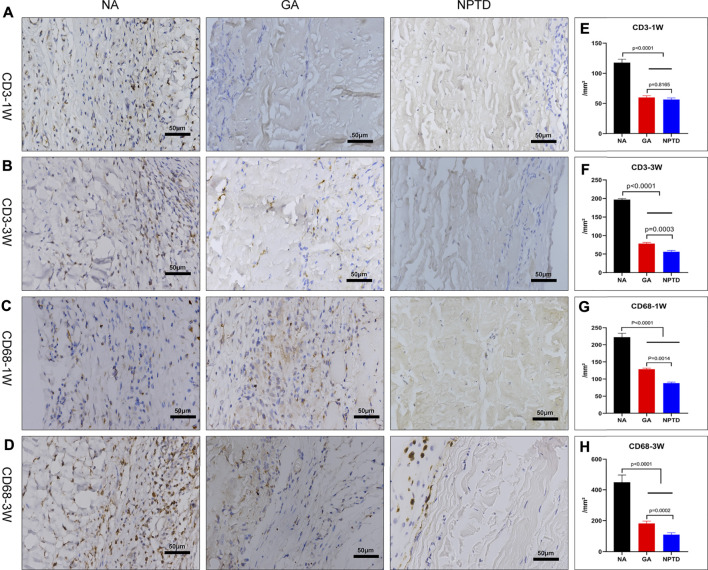
Characterization of the host response of various samples after subcutaneous implantation for 1 week and 3 weeks. The images show the immunohistochemical staining of CD68 and CD3 **(A,B,C,D)**, on the right side of the corresponding image is the semi-quantitative result **(E–H)**. In the first week, CD3^+^ cells were not significantly different between the GA and NPTD groups, while in the third week, there were fewer CD3 positive cells in the NPTD group than in the GA group. The results showed the reduced CD68^+^ inflammatory cell infiltration in the NPTD group compared to the GA and NA groups at 1 week and 3 weeks. Statistical analysis was performed by one-way analysis of variance (ANOVA).

To investigate the inflammatory response of the samples *in vivo*, the infiltration of inflammatory cells was detected at 1 week and 3 weeks after implantation (Figure 8). The NA group showed prominent infiltration of host cells. The results of IHC showed that the NA group had more CD3^+^ and CD68^+^ cells infiltrating the tissue compared to the GA and NPTD groups at 1 week and 3 weeks, indicating that a severe immune inflammatory response was triggered *in vivo*. After 3 weeks of implantation, the IHC staining of GA group revealed that there were CD3^+^ and CD68^+^ inflammatory cell infiltration in the fibrous capsule and superficial layer of the leaflets, but not infiltrate into the middle layer. Notably, After 1 week and 3 weeks of implantation, the NPTD group had limited inflammatory cell infiltration, and the number of inflammatory cells (CD3^+^ and CD68^+^) was significantly lower than that in the NA and GA groups, indicating that the activated inflammatory response was milder *in vivo*.

### 3.8 Calcification and stability of leaflets *in vivo*

**FIGURE 9:**
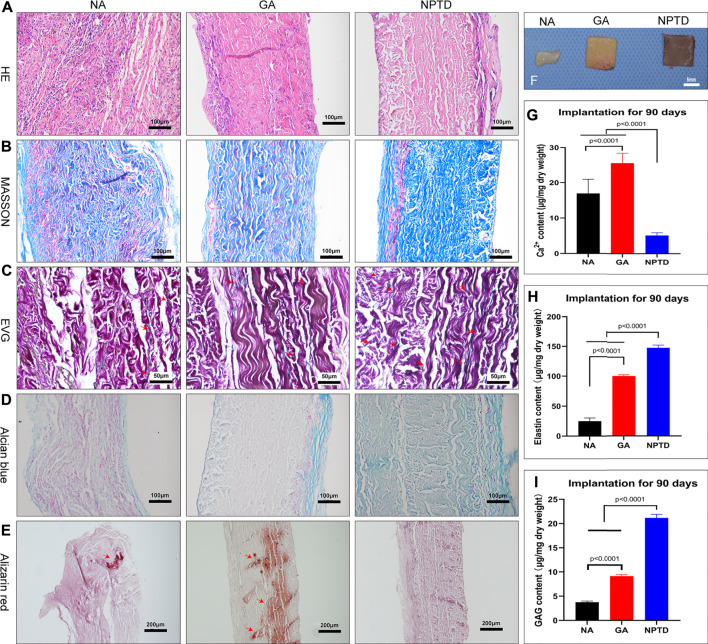
The gross specimens, Histological characteristics and quantitative analysis after subcutaneous implantation for 90 days. The pericardium in the NA group was severely degraded *in vivo*, while the pericardium in the GA and NPTD groups was well preserved **(F)**. The images show the HE staining **(A)**, Masson’s trichrome staining **(B)**, elastic Van-Gieson staining **(C)** and Alcian blue staining **(D)**, Red arrows depict the preserved elastic fibers. The Alizarin red staining show that the GA group had more calcium deposition than the NPTD group **(E)**, Red arrows depict the location of calcification. The calcium quantitative analysis showed that the calcium content of the NPTD group was significantly lower than that of the GA group **(G)**. The elastin and GAGs quantitative analysis showed that the preserved content of the NPTD group was significantly higher than that of the GA group **(H,I)**. Statistical analysis was performed by one-way analysis of variance (ANOVA).

After 90 days of implantation, the extracellular matrix of the NA group had been severely degraded, whereas the GA and NPTD groups were stable without physical deformation (Figure 9F). The HE (Figure 9A), Masson’s trichrome (Figure 9B), EVG (Figure 9C), and Alcian blue (Figure 9D) staining showed that the samples were infiltrated by a large number of cells, the structure of collagen fibers was disorganized, the elastic fibers and GAGs were broken and degraded the NA group. In contrast, in the GA and NPTD groups, the sample structure was similar to that before implantation, however, there was degradation of elastic fibers and GAGs in the GA group. The preserved elastin and GAGs content in the NPTD (148.50 ± 1.81 μg/mg, 21.18 ± 0.76 μg/mg) group was significantly higher than that in the GA (99.87 ± 1.40 μg/mg, 9.16 ± 0.32 μg/mg) and NA (25.07 ± 2.11 μg/mg, 3.76 ± 0.25 μg/mg) group (Figures 9H,I). Alizarin Red staining of the GA and NA groups showed distinct calcium deposition after 90 days of implantation, which appeared as dark-red parts, this was not observed on the NPTD group (Figure 9E). This was further confirmed with quantitative analysis of the calcium content (Figure 9-G). In the NPTD group, the calcium concentrations were (5.15 ± 0.24 μg/mg), significantly lower than that of the GA (25.56 ± 0.88 μg/mg) and NA (16.93 ± 1.28 μg/mg) groups.

## 4 Discussion

In the past decade, Glut has been used to crosslink BHV to render them stable and less immunoinflammation for implantation in clinical application (Nimni et al., 1987; Mendoza-Novelo and Cauich-RodriGuez, 2011). However, Glut crosslinked valves failed within 12–15 years after implantation (Schoen, 2008; Manji et al., 2012). Due to Glut only crosslinked adjacent amino groups to stabilize collagen fibers, while other key ECM components, such as GAGs and elastin, cannot be stably preserved after implantation. The destruction of the integrity of extracellular matrix (ECM) leads to calcification or biomechanical property damage (Sacks and Schoen, 2002; Mirnajafi et al., 2006). It should also be noted that calcification could accelerate degeneration of tissue. In addition, the drawbacks of residual toxicity, inflammatory reaction are frequently met in Glut crosslinked valves (Liu et al., 2019; Guo et al., 2021).

Elastin and GAGs, which play an important role in the biomechanics of native valve tissue, can be degraded by host and endogenous enzymes, as well as persistent mechanical fatigue of the tissue (Lee et al., 2001; Tripi and Vyavahare, 2014). GAGs are abundant in cardiac valve leaflet tissue and participate in many aspects of physiological and pathological functions. Moreover, GAGs maintain leaflet morphology, hydration, and mechanical function (Lovekamp et al., 2006). Elastin provide tissue elasticity, which provides BHV with the ability to rapidly recover to the resting position during diastole (Vesely, 1998). Perhaps retaining ECM components by appropriate chemical crosslinking will enable more prolonged stabilization and extended implant life. Previous studies showed that Glut combined with crosslinking-agents that protect elastin or GAGs, although it could reduce calcium deposition, long-term structural stabilization remains a challenge (Shah and Vyavahare, 2008; Wang et al., 2008). Thus, we innovatively employed hybrid crosslinking method (NPTD) to stabilize the three main components of bovine pericardium and improve durability of BHVs.

In our study, the hybrid crosslinking method consist of a combination of Polyethylene glycol diglycidyl ether, Neomycin trisulfate and Tannic acid. Considering the irresistible drawbacks of Glut, PE was used as an alternative to Glut and to crosslink collagen. Polyepoxy compounds treatment have been shown to impart hydrophilicity, pliability, sterility, and high calcification resistance to biomaterials (Zhuravleva et al., 2021). The epoxy group has an oxygen arm (O ═ O) that can work as a flexible joint in the cross-linking bridge, while the carbon arm of the aldehyde group is mechanically a stiff joint. In addition, there are multiple epoxy groups in PE that can react not only with amino groups but also with carboxyl groups of proteins in collagen fibrils under different pH conditions (Xi et al., 1992; Sung et al., 1996b). Neomycin is a hyaluronidase inhibitor which contains six amine functionalities and could be available for carbodiimide cross-linking to carboxyl groups of GAG molecules (Raghavan et al., 2007). TA could stabilize elastin fibers against enzymatic degradation. The mechanism may be related to the fact that polyphenols could physically interact with hydrophobic regions of elastin and block the elastase cleavage site, which is believed to resist calcification deposition (Isenburg et al., 2005). In present work, the innovative hybrid method of three excellent crosslinking-agents creates a more robust for the entire composite through an irreversible meshwork of crosslinking.

The microstructure can reflect the morphology and characteristics of the crosslinked leaflets (Wang et al., 2017). To observe the effect of the NPTD and GA crosslinking on the leaflets microstructure, histological staining and SEM were applied. The results of histological indicated that compared with the natural pericardium, the tissue structure of GA and NPTD crosslinked pericardium is more compact, and collagen and elastic fibers mesh with each other. The SEM of leaflet cross-sections showed a compact structure in the GA and NPTD groups without loose pores and surface ruffles. Futuremore, the SEM revealed that the native pericardial surface was roughness and testing of contact angles revealed low hydrophilicity. However, the pericardial surface was flat after the GA and NPTD cross-linking, and the fibers were not obviously bending had better hydrophilicity. In addition, the hydrophilicity of the NPTD group was better than that of the GA group, and perhaps the PE and TA contained more hydrophilic groups (Sung et al., 1996a; Isenburg et al., 2005). Which improved the hydrophilicity of the material, on the other hand, the modification of the roughness after cross-linking, which also improved the hydrophilicity of the material (Rupp et al., 2018). Hence, the alteration of material’s surface properties, such as surface roughness and hydrophilicity, may greatly influence the hemocompatibility on the material. Including platelet adhesion and hemolysis ratio were significantly lower in the NPTD group, indicating that it may have anti-coagulation properties *in vivo*. The Mimicking of the behavior of HUVECs is essential to evaluate the cytocompatibility and toxicity of bioprosthetic heart valves. As our results showed, both cell adhesion and proliferation tests were better in the NPTD group than that in the GA group, suggesting that the hybrid cross-linking method has the characteristics of low toxicity, which provides an opportunity for endothelialization and regeneration of BHVs *in vivo* (Guo et al., 2021).

Heart valves control unidirectional blood flow within the heart during the heart cycle which experience three distinct states of tissue stress: tension, bending, and shear stress. The collagen fibers provide the necessary tensile strength for the process of opening and closing of the leaflets. GAGs endow compressive properties to the valve and allow it to absorb stress during closure. Elastic fibers lengthen the leaflets as the valve opens and promote recoils when it closes (Ayoub et al., 2016). Valve leaflets are susceptible to strain and creep during opening and closing, resulting in an accumulation of strain localized in the high stress region of the leaflet. This strain accumulation is likely to ultimately lead to leaflet failure. Heart valves must have an extraordinary ability to withstand the harsh mechanical environment of the heart, requiring a stable extracellular matrix and excellent biomechanics to achieve life-time durability (Hasan et al., 2014; Ayoub et al., 2016). In the hybrid crosslinking method, the reaction conditions were optimized to enhance the reaction between the PE and the amino and carboxyl groups on the leaflets, strengthening the connection between the collagen fibers. Meanwhile, the treatment of TA, which can enhance not only the cross-linking of elastic fibers but also strengthens collagen and elastic fiber junctions, further improved the stability of collagen (Heijmen et al., 1997; Isenburg et al., 2004). Avoiding rapid degradation to prolong working time *in vivo* is the main function of cross-linking. Therefore, it is necessary to take some indicators to evaluate the efficiency of crosslinking while measuring the stability of the modified leaflets. The tissue thermal denaturation temperature is related to the stability of collagen (Tripi and Vyavahare, 2014), the Td was higher in the NPTD group compared to the GA group, suggesting that the hybrid crosslinking method can improve the stability of the leaflets. In addition, under the stresses experienced in a leaflet, materials with higher Young’s modulus would be more resistant to strain accumulation and, consequently, failure (Bernacca et al., 2002). Furthermore, the Young’s modulus tends to be inversely proportional to the energy loss during valve closure, so that increasing Young’s modulus reduces counter flow through the valve and improve valve closure efficiency. On the other hand, reduced leaflet thickness may also improve the closing efficiency of the valve at higher cardiac output (Bernacca et al., 2002). This indicated that crosslinked leaflets can better withstand mechanical mechanics *in vivo*, reduce energy loss and improve the durability of the valve.

Clinically, BHVs may be subjected to storage for up to 36 months. There was a significant loss of GAGs during storage with up to 30% loss of Glut cross-linked leaflets at 6 months of storage. Degradation of elastin can also occur (Friebe et al., 2011). To further verify the effectiveness of the hybrid cross-linking method, enzymatic hydrolysis experiments and storage degradation experiments *in vitro* were performed, which are often used to evaluate the stability of the cross-linked tissues. It is well known that epoxy compounds use their epoxy functional groups to fix biological tissues, and multiple epoxy groups in PE react with amino, carboxyl, and hydroxyl groups to mask and crosslink amino groups in collagen, which can strengthen the cross-linking of collagen fibers and improve the resistance to enzymes (Sung et al., 1996a; Sung et al., 1996b). Neomycin is thought to induce conformational changes by binding to hyaluronidase, rendering them non-functional and inactive (Raghavan et al., 2007). In addition, TA forms multiple hydrogen bonds with proteins, especially those rich in proline, such as elastin and collagen and improves the resistance to elastase (Isenburg et al., 2004). The results of enzymatic hydrolysis showed that the collagenase resistance of the NPTD group was similar to that of the GA group, and the loss of collagen was significantly reduced. Whereas the resistance to elastase and hyaluronidase was better in the NPTD group than in the GA and NA groups, more elastin and GAGs were retained after enzymatic hydrolysis. For the degradation experiment *in vitro*, there was no obvious degradation of collagen in the NPTD and GA groups after storage for 90 days. But the loss of elastin and GAGs in the GA group was similar to that in the NA group, whereas the loss of both was significantly lower in the NPTD group. Therefore, these all indicate that the hybrid cross-linking method has a stabilizing effect on the three main components of the leaflet and could reduce the mass loss for *in vitro* preservation and improve the resistance to enzymatic hydrolysis, which may extend the implantation life of BHVs.

As a xenograft, BHV can cause inflammatory reactions when implanted *in vivo* (Li, 2019). Macrophages and lymphocytes surrounding the implanted material were utilized to characterize the immune-inflammatory response of the leaflets, which could be specifically labeled by CD68 and CD3, respectively. (Yu et al., 2021). As the results showed, the pericardium of the NA group was infiltrated by a large number of CD68^+^ and CD3^+^ cells within 1 week and 3 weeks of implantation, indicating that the uncrosslinked xenografts could cause a severe immune-inflammatory response which may be associated with natural barrier caused by the carbohydrate antigen (Senage et al., 2022). The crosslinking is considered to be a method that can reduce the antigenicity of tissues by masking and reducing antigenic sites (Xiang et al., 2017). GA crosslinked biomaterials have been shown to cause inflammatory responses and fibrous encapsulation (Deeken et al., 2011). In our study, after 1 week and 3 weeks of subcutaneous implantation, the CD68^+^ and CD3^+^ cells were detected in the fibrous capsule and leaflet in GA group, which were significantly higher than those in the NPTD group. This may be due to the residual aldehyde group or the release of free aldehyde molecules as Schiff base bonds are degraded, which activates the host cell response (Liu et al., 2020). Furthermore, the uncrosslinked tissue components and exposed xenogenic carbohydrate antigens can also induce immune inflammatory responses (Reuven et al., 2016; Liu et al., 2020; Human et al., 2021). While the hybrid cross-linking method could further mask the antigenic sites of the tissue by cross-linking the three components in the leaflets. In addition, TA, as a plant polyphenol, has pleiotropic effects such as anti-inflammatory, antioxidant, antimicrobial properties by scavenging oxygen and oxygen derived free radicals (Isenburg et al., 2005). The results of the early inflammatory response of the crosslinked leaflets *in vivo* showed that the NPTD group had a milder immune inflammatory response than the GA and NA groups. Meanwhile, The inflammatory cells of the NA group were extensively infiltrated in the leaflet, those of the GA group were distributed in the superficial layer and fibrous capsule. In contrast, the inflammation cells of the NPTD group were almost all infiltrated within the fibrous capsule. That indicated that the hybrid cross-linked materials may better resist inflammatory cell infiltration and ingrowth. Therefore, these data clearly show that the stable cross-linking of the three main components in the NPTD group will not cause severe inflammatory response *in vivo*.

Subcutaneous implantation of leaflets in rat was carried out to test calcification and degeneration. This accumulation of calcified deposits in a rat subcutaneous model corresponds to more than 10 years of clinical implantation (Tam et al., 2015). Calcification is one of the essential contributions to the failure of BHVs *in vivo* (Schoen and Levy, 2005). The calcification stiffens the valve leaflets, subsequently leading to the poor mobility of the cusp, alter valve stress areas, and gradually progresses to valve stenosis or regurgitation. In addition, calcification of BHVs is mostly occurred in the areas of valve flexion, where deformation is maximal. This also indicate that improvement of the tenacity and anti-bend properties of biomechanical can decrease the degeneration and calcification of bioprosthetic valves (Xi et al., 1992). Previous study has demonstrated that GA crosslinking can lead to calcification of BHVs *in vivo*, possibly caused by free aldehyde groups and incomplete crosslinking. Collagen treated with GA is a strong calcium nucleating agent. (Guo et al., 2018; Guo et al., 2021). Calcification of collagen can be blocked by changing the cross-linking agent. In the results of 90 days of subcutaneous implantation, the NPTD group effectively inhibited the calcification of BHV tissues compared with the GA group, and Alizarin red staining of valves treated by the hybrid cross-linking method revealed no signs of calcification. Alizarin staining of GA group samples was intensely red, suggesting severe calcification of valve leaflets *in vivo*. In addition, the calcium content of the samples in the NPTD group was significantly lower compared with the GA group as well. Subcutaneous implant models were also used to characterize the preservation of components by histological staining and quantitative analysis. Uncrosslinked pericardium could be infiltrated and severely degraded by host cells *in vivo*. The results of histological staining and quantitative analysis showed that more elastin and GAGs were preserved in the NPTD group than in the GA group. All these proved that the hybrid crosslinking method can stabilize the main components of pericardium, reduce degradation and calcification *in vivo*, and improve the durability of BHVs.

## 5 Conclusion

In this study, we explored the feasibility of the novel non-glutaraldehyde hybrid crosslinking method for BHVs. The hybrid crosslinked leaflets exhibited superior biomechanical properties, biocompatibility, thermodynamics stability, less susceptible to enzymatic structural degradation, anti-inflammation, and anti-calcification property. Furthermore, these data suggest that the hybrid treatment technique produces a more biocompatible material than Glut treated tissue. This newly developed biomaterial may provide more durable leaflets for BHVs in future.

## Data Availability

The original contributions presented in the study are included in the article/Supplementary Material, further inquiries can be directed to the corresponding author.

## References

[B1] Auffret V. Puri R. Urena M. Chamandi C. Rodriguez-Gabella T. Philippon F. (2017). Conduction disturbances after transcatheter aortic valve replacement: Current status and future perspectives. Circulation 136 (11), 1049–1069. 10.1161/circulationaha.117.028352 28893961

[B2] Ayoub S. Ferrari G. Gorman R. C. Gorman J. H. Schoen F. J. Sacks M. S. (2016). Heart valve biomechanics and underlying mechanobiology. Compr. Physiol. 6 (4), 1743–1780. 10.1002/cphy.c150048 27783858 PMC5537387

[B3] Bernacca G. M. O'Connor B. Williams D. F. Wheatley D. J. (2002). Hydrodynamic function of polyurethane prosthetic heart valves: Influences of Young's modulus and leaflet thickness. Biomaterials 23 (1), 45–50. 10.1016/s0142-9612(01)00077-1 11762853

[B4] Bezuidenhout D. Oosthuysen A. Human P. Weissenstein C. Zilla P. (2009). The effects of cross-link density and chemistry on the calcification potential of diamine-extended glutaraldehyde-fixed bioprosthetic heart-valve materials. Biotechnol. Appl. Biochem. 54 (3), 133–140. 10.1042/ba20090101 19882764

[B5] Catterall F. Ames P. R. Isles C. (2020). Warfarin in patients with mechanical heart valves. Bmj 371, m3956. 10.1136/bmj.m3956 33060144

[B6] Deeken C. R. Melman L. Jenkins E. D. Greco S. C. Frisella M. M. Matthews B. D. (2011). Histologic and biomechanical evaluation of crosslinked and non-crosslinked biologic meshes in a porcine model of ventral incisional hernia repair. J. Am. Coll. Surg. 212 (5), 880–888. 10.1016/j.jamcollsurg.2011.01.006 21435917 PMC3782991

[B7] Deshmukh A. Deshmukh K. Nimni M. E. (1971). Synthesis of aldehydes and their interactions during the *in vitro* aging of collagen. Biochemistry 10 (12), 2337–2342. 10.1021/bi00788a025 5000451

[B8] Emmert M. Y. Schmitt B. A. Loerakker S. Sanders B. Spriestersbach H. Fioretta E. S. (2018). Computational modeling guides tissue-engineered heart valve design for long-term *in vivo* performance in a translational sheep model. Sci. Transl. Med. 10 (440), eaan4587. 10.1126/scitranslmed.aan4587 29743347

[B9] Fioretta E. S. Dijkman P. E. Emmert M. Y. Hoerstrup S. P. (2018). The future of heart valve replacement: Recent developments and translational challenges for heart valve tissue engineering. J. Tissue Eng. Regen. Med. 12 (1), e323–e335. 10.1002/term.2326 27696730

[B10] Friebe V. M. Mikulis B. Kole S. Ruffing C. S. Sacks M. S. Vyavahare N. R. (2011). Neomycin enhances extracellular matrix stability of glutaraldehyde crosslinked bioprosthetic heart valves. J. Biomed. Mat. Res. 99 (2), 217–229. 10.1002/jbm.b.31889 21714085

[B11] Guo G. Jin L. Jin W. Chen L. Lei Y. Wang Y. (2018). Radical polymerization-crosslinking method for improving extracellular matrix stability in bioprosthetic heart valves with reduced potential for calcification and inflammatory response. Acta Biomater. 82, 44–55. 10.1016/j.actbio.2018.10.017 30326277

[B12] Guo G. Jin L. Wu B. He H. Yang F. Xu L. (2021). A method for simultaneously crosslinking and functionalizing extracellular matrix-based biomaterials as bioprosthetic heart valves with enhanced endothelialization and reduced inflammation. Acta Biomater. 119, 89–100. 10.1016/j.actbio.2020.10.029 33099025

[B13] Hasan A. Ragaert K. Swieszkowski W. Selimović S. Paul A. Camci-Unal G. (2014). Biomechanical properties of native and tissue engineered heart valve constructs. J. Biomech. 47 (9), 1949–1963. 10.1016/j.jbiomech.2013.09.023 24290137

[B14] Heijmen F. H. du Pont J. S. Middelkoop E. Kreis R. W. Hoekstra M. J. (1997). Cross-linking of dermal sheep collagen with tannic acid. Biomaterials 18 (10), 749–754. 10.1016/s0142-9612(96)00202-5 9158858

[B15] Hu M. Peng X. Zhao Y. Yu X. Cheng C. Yu X. A.-O. (2021). Dialdehyde pectin-crosslinked and hirudin-loaded decellularized porcine pericardium with improved matrix stability, enhanced anti-calcification and anticoagulant for bioprosthetic heart valves. Biomater. Sci. 9, 7617–7635. 10.1039/D1BM01297E 34671797

[B16] Human P. Bezuidenhout D. Aikawa E. Zilla P. (2021). Residual bioprosthetic valve immunogenicity: Forgotten, not lost. Front. Cardiovasc. Med. 8, 760635. 10.3389/fcvm.2021.760635 35059444 PMC8764456

[B17] Isenburg J. C. Simionescu D. T. Vyavahare N. R. (2004). Elastin stabilization in cardiovascular implants: Improved resistance to enzymatic degradation by treatment with tannic acid. Biomaterials 25 (16), 3293–3302. 10.1016/j.biomaterials.2003.10.001 14980424

[B18] Isenburg J. C. Simionescu D. T. Vyavahare N. R. (2005). Tannic acid treatment enhances biostability and reduces calcification of glutaraldehyde fixed aortic wall. Biomaterials 26 (11), 1237–1245. 10.1016/j.biomaterials.2004.04.034 15475053

[B19] Kumar K. Azordegan N. Durston M. Li Y. Fischer G. Moghadasian M. (2013). HMG-COA reductase inhibitors do not attenuate the inflammatory response associated with glutaraldehyde-fixed bioprosthetic heart valve conduits. Can. J. Cardiol. 29 (10), S261. 10.1016/j.cjca.2013.07.429

[B20] Lee T. C. Midura R. J. Hascall V. C. Vesely I. (2001). The effect of elastin damage on the mechanics of the aortic valve. J. Biomech. 34 (2), 203–210. 10.1016/s0021-9290(00)00187-1 11165284

[B21] Leong J. Munnelly A. Liberio B. Cochrane L. Vyavahare N. (2013). Neomycin and carbodiimide crosslinking as an alternative to glutaraldehyde for enhanced durability of bioprosthetic heart valves. J. Biomater. Appl. 27 (8), 948–960. 10.1177/0885328211430542 22207605

[B22] Li K. Y. C. (2019). Bioprosthetic heart valves: Upgrading a 50-year old technology. Front. Cardiovasc. Med. 6, 47. 10.3389/fcvm.2019.00047 31032263 PMC6470412

[B23] Lim G. B. (2018). Hypertension: Lifestyle offsets genetic risk of hypertension. Nat. Rev. Cardiol. 15 (4), 196. 10.1038/nrcardio.2018.15 29493574

[B24] Liu C. Qiao W. Cao H. Dai J. Li F. Shi J. (2020). A riboflavin-ultraviolet light A-crosslinked decellularized heart valve for improved biomechanical properties, stability, and biocompatibility. Biomater. Sci. 8 (9), 2549–2563. 10.1039/c9bm01956a 32226995

[B25] Liu J. Jing H. Qin Y. Li B. Sun Z. Kong D. A.-O. (2019). Nonglutaraldehyde fixation for off the shelf decellularized bovine pericardium in anticalcification cardiac valve applications. ACS Biomater. Sci. Eng. 5 (3), 1452–1461. 10.1021/acsbiomaterials.8b01311 33405620

[B26] Liu Y. Chen C. Xie X. Yuan H. Tang Z. Qian T. (2022). Photooxidation and pentagalloyl glucose cross-linking improves the performance of decellularized small-diameter vascular xenograft *in vivo* . Front. Bioeng. Biotechnol. 10, 816513. 10.3389/fbioe.2022.816513 35402413 PMC8987116

[B27] Lohre J. M. Baclig L. Wickham E. Guida S. Farley J. Thyagarajan K. (1993). Evaluation of epoxy ether fixed bovine arterial grafts for mutagenic potential. Asaio J. 39 (2), 106–113. 10.1097/00002480-199339020-00007 8324256

[B28] Lopez-Moya M. Melgar-Lesmes P. Kolandaivelu K. de la Torre Hernández J. M. Edelman E. R. Balcells M. (2018). Optimizing glutaraldehyde-fixed tissue heart valves with chondroitin sulfate hydrogel for endothelialization and shielding against deterioration. Biomacromolecules 19 (4), 1234–1244. 10.1021/acs.biomac.8b00077 29539266 PMC6198652

[B29] Lovekamp J. J. Simionescu D. T. Mercuri J. J. Zubiate B. Sacks M. S. Vyavahare N. R. (2006). Stability and function of glycosaminoglycans in porcine bioprosthetic heart valves. Biomaterials 27 (8), 1507–1518. 10.1016/j.biomaterials.2005.08.003 16144707 PMC2262164

[B30] Manji R. A. Menkis A. H. Ekser B. Cooper D. K. (2012). Porcine bioprosthetic heart valves: The next generation. Am. Heart J. 164 (2), 177–185. 10.1016/j.ahj.2012.05.011 22877802

[B31] Mendoza-Novelo B. Cauich-Rodri Guez J. V. (2011). Decellularization, stabilization and functionalization of collagenous tissues used as cardiovascular biomaterials. Biomaterials - Physics and Chemistry. London: InTech. 978-953-307-418-4.

[B32] Mercuri J. J. Lovekamp J. J. Simionescu D. T. Vyavahare N. R. (2007). Glycosaminoglycan-targeted fixation for improved bioprosthetic heart valve stabilization. Biomaterials 28 (3), 496–503. 10.1016/j.biomaterials.2006.09.005 17030363

[B33] Mirnajafi A. Raymer J. M. McClure L. R. Sacks M. S. (2006). The flexural rigidity of the aortic valve leaflet in the commissural region. J. Biomech. 39 (16), 2966–2973. 10.1016/j.jbiomech.2005.10.026 16360160

[B34] Mohammadi H. Mequanint K. (2011). Prosthetic aortic heart valves: Modeling and design. Med. Eng. Phys. 33 (2), 131–147. 10.1016/j.medengphy.2010.09.017 20971672

[B35] Nimni M. E. Cheung D. Strates B. Kodama M. Sheikh K. (1987). Chemically modified collagen: A natural biomaterial for tissue replacement. J. Biomed. Mat. Res. 21 (6), 741–771. 10.1002/jbm.820210606 3036880

[B36] Qi X. Jiang Z. Song M. Tang Z. Xie X. Liu Y. (2022). A novel crosslinking method for improving the anti-calcification ability and extracellular matrix stability in transcatheter heart valves. Front. Bioeng. Biotechnol. 10, 909771. 10.3389/fbioe.2022.909771 35903798 PMC9315440

[B37] Raghavan D. Simionescu D. T. Vyavahare N. R. (2007). Neomycin prevents enzyme-mediated glycosaminoglycan degradation in bioprosthetic heart valves. Biomaterials 28 (18), 2861–2868. 10.1016/j.biomaterials.2007.02.017 17353047 PMC2262162

[B38] Reuven E. M. Leviatan Ben-Arye S. Marshanski T. Breimer M. E. Yu H. Fellah-Hebia I. (2016). Characterization of immunogenic Neu5Gc in bioprosthetic heart valves. Xenotransplantation 23, 381–392. 10.1111/xen.12260 27610947 PMC5036590

[B39] Rupp F. Liang L. Geis-Gerstorfer J. Scheideler L. Hüttig F. (2018). Surface characteristics of dental implants: A review. Dent. Mat. 34 (1), 40–57. 10.1016/j.dental.2017.09.007 29029850

[B40] Sacks M. S. David Merryman W. Schmidt D. E. (2009). On the biomechanics of heart valve function. J. Biomech. 42 (12), 1804–1824. 10.1016/j.jbiomech.2009.05.015 19540499 PMC2746960

[B41] Sacks M. S. Schoen F. J. (2002). Collagen fiber disruption occurs independent of calcification in clinically explanted bioprosthetic heart valves. J. Biomed. Mat. Res. 62 (3), 359–371. 10.1002/jbm.10293 12209921

[B42] Schoen F. J. (1997). Aortic valve structure-function correlations: Role of elastic fibers no longer a stretch of the imagination. J. Heart Valve Dis. 6 (1), 1–6. 9044068

[B43] Schoen F. J. (2008). Evolving concepts of cardiac valve dynamics: The continuum of development, functional structure, pathobiology, and tissue engineering. Circulation 118 (18), 1864–1880. 10.1161/circulationaha.108.805911 18955677

[B44] Schoen F. J. Levy R. J. (2005). Calcification of tissue heart valve substitutes: Progress toward understanding and prevention. Ann. Thorac. Surg. 79 (3), 1072–1080. 10.1016/j.athoracsur.2004.06.033 15734452

[B45] Senage T. Paul A. A.-O. Le Tourneau T. Fellah-Hebia I. Vadori M. Bashir S. (2022). The role of antibody responses against glycans in bioprosthetic heart valve calcification and deterioration. Nat. Med. 28, 283–294. 10.1038/s41591-022-01682-w 35177855 PMC8863575

[B46] Shah S. R. Vyavahare N. R. (2008). The effect of glycosaminoglycan stabilization on tissue buckling in bioprosthetic heart valves. Biomaterials 29 (11), 1645–1653. 10.1016/j.biomaterials.2007.12.009 18199477 PMC2268977

[B47] Sivaraman B. Latour R. A. (2010). The relationship between platelet adhesion on surfaces and the structure versus the amount of adsorbed fibrinogen. Biomaterials 315, 832–839. 10.1016/j.biomaterials.2009.10.008 PMC2790000 19850334

[B48] Sung H. W. Hsu C. S. Lee Y. S. Lin D. S. (1996). Crosslinking characteristics of an epoxy-fixed porcine tendon: Effects of pH, temperature, and fixative concentration. J. Biomed. Mat. Res. 31 (4), 511–518. 10.1002/(sici)1097-4636(199608)31:4<511::aid-jbm11>3.0.co;2-j 8836848

[B49] Sung H. W. Hsu C. S. Wang S. P. Hsu H. L. (1997). Degradation potential of biological tissues fixed with various fixatives: An *in vitro* study. J. Biomed. Mat. Res. 35 (2), 147–155. 10.1002/(sici)1097-4636(199705)35:2<147::aid-jbm2>3.0.co;2-n 9135163

[B50] Sung H. W. Hsu H. L. Shih C. C. Lin D. S. (1996). Cross-linking characteristics of biological tissues fixed with monofunctional or multifunctional epoxy compounds. Biomaterials 17 (14), 1405–1410. 10.1016/0142-9612(96)87282-6 8830967

[B51] Tam H. Zhang W. Feaver K. R. Parchment N. Sacks M. S. Vyavahare N. (2015). A novel crosslinking method for improved tear resistance and biocompatibility of tissue based biomaterials. Biomaterials 66, 83–91. 10.1016/j.biomaterials.2015.07.011 26196535 PMC4522354

[B52] Tripi D. R. Vyavahare N. R. (2014). Neomycin and pentagalloyl glucose enhanced cross-linking for elastin and glycosaminoglycans preservation in bioprosthetic heart valves. J. Biomater. Appl. 28 (5), 757–766. 10.1177/0885328213479047 24371208 PMC4594843

[B53] Vesely I. (1998). The role of elastin in aortic valve mechanics. J. Biomech. 31 (2), 115–123. 10.1016/s0021-9290(97)00122-x 9593204

[B54] Vyavahare N. Ogle M. Schoen F. J. Zand R. Gloeckner D. C. Sacks M. (1999). Mechanisms of bioprosthetic heart valve failure: Fatigue causes collagen denaturation and glycosaminoglycan loss. J. Biomed. Mat. Res. 46 (1), 44–50. 10.1002/(sici)1097-4636(199907)46:1<44::aid-jbm5>3.0.co;2-d 10357134

[B55] Wang D. Jiang H. Li J. Zhou J. Y. Hu S. S. (2008). Mitigated calcification of glutaraldehyde-fixed bovine pericardium by tannic acid in rats. Chin. Med. J. 121 (17), 1675–1679. 10.1097/00029330-200809010-00017 19024098

[B56] Wang X. Wen K. Yang X. Li L. Yu X. (2017). Biocompatibility and anti-calcification research of a biological artery fixed with natural-occurred phytic acid as crosslinking reagent. 10.1039.C7TB02090B. 10.1039/c7tb02090b 32264650

[B57] Xi T. Liu F. Xi B. (1992). Effect of pretreatment with epoxy compounds on the mechanical properties of bovine pericardial bioprosthetic materials. J. Biomater. Appl. 7 (1), 61–75. 10.1177/088532829200700104 1432579

[B58] Xiang J. Liu P. Zheng X. Dong D. Fan S. Dong J. (2017). The effect of riboflavin/UVA cross-linking on anti-degeneration and promoting angiogenic capability of decellularized liver matrix. J. Biomed. Mat. Res. A 105 (10), 2662–2669. 10.1002/jbm.a.36126 28556592

[B59] Xu S. Lu F. Cheng L. Li C. Zhou X. Wu Y. (2017). Preparation and characterization of small-diameter decellularized scaffolds for vascular tissue engineering in an animal model. Biomed. Eng. OnLine 16, 55. 10.1186/s12938-017-0344-9 28494781 PMC5425976

[B60] Yu T. Yang W. Zhuang W. Tian Y. Kong Q. Chen X. (2021). A bioprosthetic heart valve cross-linked by a non-glutaraldehyde reagent with improved biocompatibility, endothelialization, anti-coagulation and anti-calcification properties. J. Mat. Chem. B 9 (19), 4031–4038. 10.1039/d1tb00409c 33908590

[B61] Zhang R. Wang Y. Chen L. Wang R. Li C. Li X. (2018). Reducing immunoreactivity of porcine bioprosthetic heart valves by genetically-deleting three major glycan antigens, GGTA1/β4GalNT2/CMAH. Acta Biomater. 72, 196–205. 10.1016/j.actbio.2018.03.055 29631050

[B62] Zhuravleva I. Y. Karpova E. V. Oparina L. A. Poveschenko O. V. Surovtseva M. A. Titov A. T. (2021). Cross-linking method using pentaepoxide for improving bovine and porcine bioprosthetic pericardia: A multiparametric assessment study. Mater. Sci. Eng. C 118, 111473. 10.1016/j.msec.2020.111473 33255052

[B63] Zilla P. Brink J. Human P. Bezuidenhout D. (2008). Prosthetic heart valves: Catering for the few. Biomaterials 29 (4), 385–406. 10.1016/j.biomaterials.2007.09.033 17950840

